# Examining cognition and brain networks using magnetoencephalography in paediatric autoimmune encephalitis and acute disseminated encephalomyelitis: a preliminary study

**DOI:** 10.1093/braincomms/fcae248

**Published:** 2024-08-08

**Authors:** Charly H A Billaud, Amanda G Wood, Daniel Griffiths-King, Klaus Kessler, Evangeline Wassmer, Elaine Foley, Sukhvir K Wright

**Affiliations:** Institute of Health and Neurodevelopment and College of Health and Life Sciences, Aston University, Birmingham B4 7ET, UK; Department of Psychology, School of Social Sciences, Nanyang Technological University, Singapore 639798, Singapore; Institute of Health and Neurodevelopment and College of Health and Life Sciences, Aston University, Birmingham B4 7ET, UK; School of Psychology, Deakin University, Melbourne, Victoria 3125, Australia; Institute of Health and Neurodevelopment and College of Health and Life Sciences, Aston University, Birmingham B4 7ET, UK; Institute of Health and Neurodevelopment and College of Health and Life Sciences, Aston University, Birmingham B4 7ET, UK; School of Psychology, University College Dublin, Dublin 4, Ireland; Institute of Health and Neurodevelopment and College of Health and Life Sciences, Aston University, Birmingham B4 7ET, UK; Department of Neurology, Birmingham Women’s and Children’s Hospital, Birmingham B4 6NH, UK; Institute of Health and Neurodevelopment and College of Health and Life Sciences, Aston University, Birmingham B4 7ET, UK; Institute of Health and Neurodevelopment and College of Health and Life Sciences, Aston University, Birmingham B4 7ET, UK; Department of Neurology, Birmingham Women’s and Children’s Hospital, Birmingham B4 6NH, UK

**Keywords:** neurodevelopment, acute disseminated encephalomyelitis (ADEM), autoimmune encephalitis, magnetoencephalography (MEG), NMDAR-Ab encephalitis

## Abstract

Paediatric autoimmune encephalitis, including acute disseminated encephalomyelitis, are inflammatory brain diseases presenting with cognitive deficits, psychiatric symptoms, seizures, MRI and EEG abnormalities. Despite improvements in disease recognition and early immunotherapy, long-term outcomes in paediatric autoimmune encephalitis remain poor. Our aim was to understand functional connectivity changes that could be associated with negative developmental outcomes across different types of paediatric autoimmune encephalitis using magnetoencephalography. Participants were children diagnosed with paediatric autoimmune encephalitis at least 18 months before testing and typically developing children. All completed magnetoencephalography recording at rest, T_1_ MRI scans and neuropsychology testing. Brain connectivity (specifically in delta and theta) was estimated with amplitude envelope correlation, and network efficiency was measured using graph measures (global efficiency, local efficiency and modularity). Twelve children with paediatric autoimmune encephalitis (11.2 ± 3.5 years, interquartile range 9 years; 5M:7F) and 12 typically developing controls (10.6 ± 3.2 years, interquartile range 7 years; 8M:4F) participated. Children with paediatric autoimmune encephalitis did not differ from controls in working memory (*t*(21) = 1.449; *P =* 0.162; *d* = 0.605) but had significantly lower processing speed (*t*(21) = 2.463; *P =* 0.023; Cohen’s *d =* 1.028). Groups did not differ in theta network topology measures. The paediatric autoimmune encephalitis group had a significantly lower delta local efficiency across all thresholds tested (*d =* −1.60 at network threshold 14%). Theta modularity was associated with lower working memory (*β =* −0.781; *t*(8) = −2.588, *P =* 0.032); this effect did not survive correction for multiple comparisons (*P*(corr) *=* 0.224). Magnetoencephalography was able to capture specific network alterations in paediatric autoimmune encephalitis patients. This preliminary study demonstrates that magnetoencephalography is an appropriate tool for assessing children with paediatric autoimmune encephalitis and could be associated with cognitive outcomes.

## Teaser

Paediatric autoimmune encephalitis and acute disseminated encephalomyelitis are neurological disorders that can cause long-term cognitive problems. Billaud *et al*. used magnetoencephalography to investigate brain network changes and report alterations in affected children present many years following the acute illness, which may be associated with working memory.

## Graphical Abstract

**Graphical Abstract:**
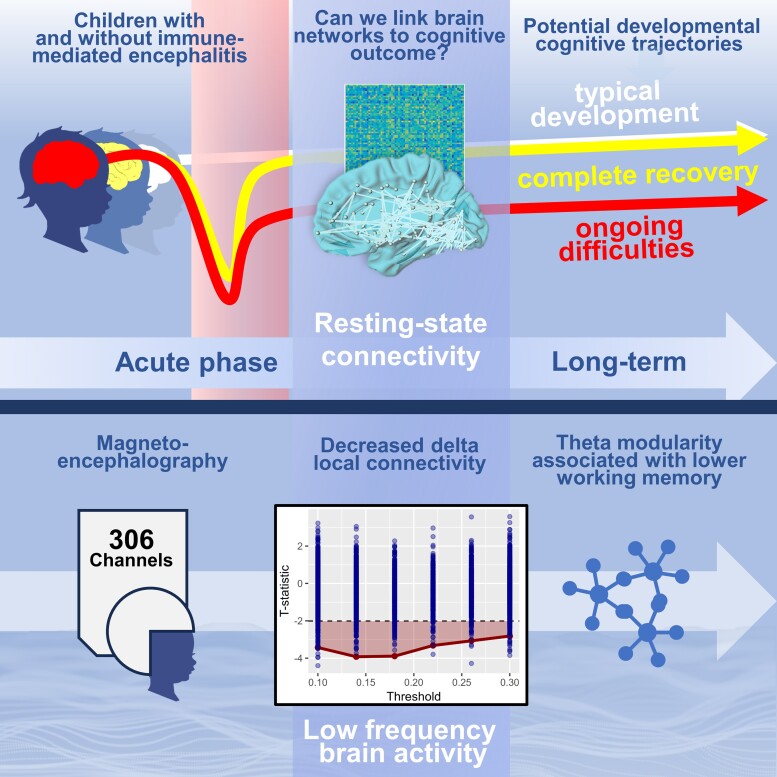

## Introduction

Paediatric autoimmune encephalitis (PAE) is an inflammatory brain disease associated with seizures, movement disorders, neuropsychiatric symptoms and cognitive deficits.^1,2^ Despite advances in diagnosing and treating PAE, a proportion of children are still left with long-term cognitive and academic difficulties [up to 45% in *N*-methyl-D-aspartate receptor antibody encephalitis (NMDARE)^3-5^ and 15.8–22% in acute disseminated encephalomyelitis (ADEM)].^6,7^ Routinely used clinical outcome assessments such as the modified Rankin Scale (mRS) or the extended disability status scale focus on motor abilities missing independent subtle cognitive deficits.^4,7-9^ A recent review in adults with autoimmune encephalitis (AE) found the most frequent impairments were found in tests of visual and verbal learning/memory, processing speed, attention and executive functions.^10^

Functional connectivity is an indicator of neural activity that reveals a systematic relationship between brain areas and is therefore interpreted as reflecting neural connections. Analyses of the spontaneous brain activity in the absence of any task (‘resting state’) offer great potential for investigating the underlying mechanisms of cognitive sequelae in AE. In adults, resting-state functional MRI (fMRI) studies suggest that AE patients have reduced functional connectivity.^11^ Altered connectivity (i.e. ‘dysconnectivity’) may reflect disruptions in neural connections, affecting cognitive functions that rely on the former. Network dysconnectivity in adult AE has been linked to lower cognitive performance,^11-14^ disease severity^11,12^ and psychiatric symptoms.^11^ Specifically, deficits in working memory and information processing speed correlated with functional connectivity.^14^ To date, children and young people with AE have not been specifically investigated for functional dysconnectivity, despite evidence that they may be more adversely affected neuropsychologically,^3,15^ partly due to the developing brain’s vulnerability.^16^ It is therefore imperative that changes in functional connectivity that could be associated with negative developmental outcomes are investigated in PAE.

Magnetoencephalography (MEG) is an alternative non-invasive method used for measuring functional brain dynamics, but studies in PAE are limited to case reports.^17,18^ MEG provides a direct measure of neuronal activity, unlike fMRI, which relies on a surrogate signal based on blood oxygenation levels, and can reveal changes in functional connectivity with higher temporal resolution than fMRI.^19^ In addition, MEG provides a better spatial resolution than EEG, being less susceptible to the spatial distortions due to tissue layers surrounding the brain with variable electric conductivity. Resting-state MEG recordings can be used to infer functional networks,^20^ in a similar way as fMRI.^11^ MEG has been widely used in paediatric epilepsy as a correlate of cognitive outcomes^21^ with the same advantages as EEG (cost-effectiveness, non-invasiveness and reproducibility).^22^ In addition, MEG is practically preferable for children, requiring less preparation time than EEG,^22^ and avoiding long periods in a noisy environment like fMRI.

EEG is abnormal in >95% of adult and paediatric AE patients showing encephalopathy, and changes in specific frequency bands such as altered theta–delta activity, extreme delta brushes and generalized rhythmic delta activity are reported.^23-25^ Theta brain networks are linked to attention and stimulus processing, encoding and consolidation of information in memory and executive functions including working memory.^26^ Delta networks are also associated with attention and concentration and working memory.^27^

In this study, we investigated whether MEG-derived delta and theta connectivity in PAE was associated with cognitive measures. We hypothesized that following AE, children would have cognitive dysfunction, and changes in resting-state networks compared with typically developing controls. We further hypothesized that these network measures would be associated with lower cognitive performance in PAE.

## Materials and methods

### Participants

Patients were recruited between 2018 and 2022 from Birmingham Children’s Hospital, Birmingham, UK, as part of a larger study investigating the effect of neurological disease on the developing brain (Aston University Ethics reference #18/LO/0990; #IRAS 233424). Healthy, typically developing controls were recruited from the local community through social media advertisement and local outreach events (Aston University Ethics reference #HLS21011). Consent was obtained according to the Declaration of Helsinki and approved by ethical committees of each institution.

#### Inclusion criteria for patients

Diagnosis of immune-mediated encephalitis, according to established criteria^1^ at least 18 months after disease onset, aged 6–16 years at the time of recruitment and cognitive assessment. Exclusion criteria included dissent of the child from participating and/or contraindications for MRI scanning. For typically developing controls, exclusion criteria included a diagnosis of learning difficulty, psychiatric, neurodevelopmental, neurological disorder, a known or suspected cerebral abnormality, dissent of the child from participating and/or contraindications for MRI scanning.

Information regarding the children’s clinical and disease course were collected from medical records by paediatric neurologists (S.K.W. and E.W.) including the mRS^28^ assessment to classify the disability in patients (score of 0 = no disability; 3 = moderate disability requiring some help but able to walk; 5 = severe disability requiring constant care for all needs; 6 = death).

### Neuropsychological assessment

The ‘Wechsler Intelligence Scale for Children, 5th Edition’ (WISC-V) was used to assess general intellectual functioning in all children.^29^ The Wechsler scale uses a battery of psychometric tests with separate indices. Working memory and processing speed indices were selected for this study analysis as these are the most frequently studied and recommended in AE.^3,10^

### MRI image acquisition

Each participant underwent structural MRI scans (T1w MPRAGE and T2-FLAIR), acquired using a 3T MRI scanner located at the Aston Institute of Health and Neurodevelopment, Birmingham, UK. Till March 2022, the MRI scanner was upgraded from a Siemens TrioTim [T_1_ parameters: TE (ms) = 3.37; TR (ms) = 1900; flip angle = 15; dimensions = 176 × 240 × 256. T2-FLAIR parameters: TE (ms) = 392; TR (ms) = 5000; flip angle = 150; dimensions = 176 × 250 × 250]. From March 2022, the scanner was upgraded to a Siemens MAGNETOM Prisma [T_1_ parameters: TE (ms) = 3.41; TR (ms) = 1900; flip angle = 15; dimensions = 176 × 240 × 256. T2-FLAIR parameters: TE (ms) = 282; TR (ms) = 5000; flip angle = 150; dimensions = 176 × 240 × 240]. Three children with AE and all controls were scanned using the newer Prisma MRI.

Preprocessing of structural MRI (T1w MPRAGE) was done with FreeSurfer (v6.0):^30^ including segmentation of grey matter, white matter and CSF boundaries; brain extraction; normalization and automated structural parcellation.^31^ Intensity correction was done with non-parametric non-uniform intensity normalization.^30^ White and grey matter surfaces were generated, smoothed and aligned.^31^ Pial surface estimation was improved with the contrasts from the FLAIR scans when available (‘-FLAIRpial’). Every scan was visually quality-checked for skull-stripping and segmentation errors: manual edits were done in FreeSurfer to remove artefacts, remaining unwanted tissues and surface estimation errors. Scans with excessive artefacts after visual inspection were discarded (one PAE and one control).

### MEG protocol and processing

#### MEG acquisition parameters

MEG recordings were conducted using the Elekta Neuromag® TRIUX MEG system at the Aston Institute of Health and Neurodevelopment, comprising 306-channels (102 magnetometers and 204 planar gradiometers) in a single-shell magnetically shielded room with MaxShield™ technology. Sampling rate = 2000 Hz; high-pass filter = 0.1 Hz and low-pass filter = 330 Hz. Internal active shielding was off. Head position indicator coils were placed on the participant, three on the forehead and one on each mastoid to record head movements. Coregistration between MEG and MRI scans was facilitated using a ‘Polhemus Fastrak’ motion tracker, which digitizes the coordinates of each participant’s head shape. Due to the fixed helmet size of the Elekta Neuromag® Triux wholehead system and the smaller paediatric head circumference of the current cohort, there was space for children to move their heads within the helmet during recordings. To mitigate this, the study team used the available ‘advanced patient positioning system’ (inclusive of paediatrically optimized cushion set), to ensure full contact of the helmet with the very top of the head, with cushion support around the seat to minimize body movement. All staff doing MEG recordings for the current study was trained specifically to carry out paediatric MEG recordings.

#### Resting-state protocol

MEG recordings were done ‘at rest’, in which participant were instructed to sit still in the scanner for 6–6:30 min and to look at a black fixation cross projected on a white background.

#### MEG coregistration and source modelling

Coregistration was done using BrainStorm (*v*3.210818, 18 August 2021),^32^ which is documented and freely available for download online under the GNU general public license (http://neuroimage.usc.edu/brainstorm), using fiducial points manually defined on the T1w MRI and the head position coordinates. 3D brain models were imported from the FreeSurfer pipeline and down sampled to 15 000 vertices. If models were unavailable (e.g. too many artefacts to generate surfaces or preprocess), an age-matched paediatric symmetric MRI template^33,34^ preprocessed with the same FreeSurfer pipeline was used as a substitute (needed for one PAE and one control participant). Three-shell realistically shaped head models were generated with adaptive integration in ‘BrainStorm’ using the ‘OpenMEEG’ plug-in^35,36^ with scalp/skull/brain layers and default settings, and the boundary element method.^37^ Signal sources were reconstructed in each brain model using LCMV beamformer, which suppresses external background noise captured in a data covariance matrix extracted from each recording, regularized with the median eigenvalues.

### Resting-state network analysis

Resting-state recordings were epoched into non-overlapping 10 s epochs using *Fieldtrip* (*v*22.01.2021).^38^ Signals were filtered with a fourth-order Butterworth zero-phase low-pass filter (cut-off: 70 Hz, as in).^39^ An independent component analysis (ICA) was used (*N* components = *N* included channels), to reject heartbeats, eye blinks and eye movement artefacts. Each individual trial was manually inspected to reject trials and channels containing excessive muscle activity and SQUID jumps artefacts. The data were imported into *BrainStorm* (*v*3.210818, 18 August 2021) for connectivity analysis. Epochs that remained were distributed as follows: 37.5 ± 1.6 [interquartile range (IQR) = 1] for PAE and 37.33 ± 1.1 (IQR = 3) for controls.

Weighted averages of the epochs were transformed in the time-frequency domain using Hilbert transformation. Frequency bands of interest were delta (1–4 Hz) and theta (5–8 Hz). Using the Desikan–Killiany atlas, Brainstorm’s scout function produced the mean time-frequency signal within each region before estimating frequency connectivity. Connectivity was computed using amplitude envelope correlation, a measure of temporal evolution of spectral power (envelope), correlated between pairs of orthogonalized signals within separate regions.^40^ Two 68 × 68 connectivity matrices were generated for each epoch and averaged; resulting in one average delta (example given in Supplementary Fig. 1) and theta connectivity matrices per participant.

The connectivity matrices were exported in Matlab, negative correlations set to zeros and the Brain Connectivity Toolbox (BCT *v*2019-03-03)^40^ was used to normalize the matrices’ weights into a range from 0 (no connection) to 1 (maximum connectivity). Proportional thresholds were chosen for their higher graph metrics stability; keeping 10–30% strongest connections for each matrix (as in previous research),^41^ in intervals of 4%: 6 matrices per frequency band. Measures of efficiency and modularity that reflect how well brain networks integrate and segregate distinct modules to efficiently transmit neuronal information, were investigated as they were applied in previous AE fMRI research:^42,43^

- Modularity (*M*), the degree to which the network can be subdivided into non-overlapping groups^40^ (using *Q* maximized modularity).
- Global efficiency (*E*_glob_), the average inverse shortest path length between all pairs of nodes.^40^
- Mean local efficiency (*E*_loc_), the global efficiency computed for neighbouring nodes at the level of each node^40^ (with recommended 2017 BCT function).

### Statistical analyses

#### Multi-threshold permutation correction for network selection

To mitigate multiple comparison biases, multi-threshold permutation correction (MTPC) was applied, which identifies stable group differences across thresholds, using the ‘brainGRAPH’ package (*v*3.0.0) in *R* (*v*4.2.1; R Core Team, 2022). The brainGRAPH Desikan–Killiany atlas label order was matched to the Brainstorm matrices. A *t*-test-statistic was computed for graph measures across groups at each threshold, permuted 5000 times. The maximum *t* across permutations was used to establish a critical *t*-value at *α* = 0.05. For each threshold, an area-under-the-curve (AUC) was computed for significant ‘clusters’ where observed *t* > critical value. A critical AUC is determined from the mean of the AUCs above critical value: the difference is significant if the AUC of the significant clusters exceeds the critical AUC. The model was one-sided and tested reduced connectivity in PAE.

The *P*-values corrected across thresholds in each MTPC (p.fdr), were corrected again for false discovery rate against the p.fdr of the other MTPC analyses. For significant differences, *post hoc* analyses also checked for difference in overall raw functional connectivity (Supplementary Material 1).

#### Multiple regressions for resting-state networks

Graph measures were extracted for the thresholds where the group difference was the highest and included as independent variables in multiple regression analyses with processing speed index and working memory index (WMI) as dependant variables, controlling for sex. Age was not controlled for as the standard WISC scores are already standardized across age-range.

## Results

### Demographics

Twenty-four children completed the study, including 12 PAE patients and 12 typically developing children (Table 1). In PAE, the average time from disease onset to scanning was 7.3 years (3–15 years). Age at time of MEG ranged from 6 years and 2 months to 15 years and 9 months. Nine were diagnosed with ADEM (four serum MOG-antibody positive), and three with AE (one with NMDAR-antibodies, one with MOG-antibodies and one antibody-negative). Three had comorbid neurodevelopmental diagnoses, which were diagnosed after the initial AE/ADEM insult; none were diagnosed or raised as a concern prior to this. One had diagnoses of autism spectrum disorder (ASD), attention deficit hyperactivity disorder, dyspraxia and focal epilepsy; one had epilepsy and one had a diagnosis of ASD. Four were on antiseizure medications. The average Modified Rankin score for the PAE patients was 1.0 (range 0–3).

**Table 1:** Comparison of children with AE and typically developing children

Child characteristics	Controls (*N* = 12)	AE (*N* = 12)	Statistics	
			Test	*P*-value
Sex (female:male)	4:8	7:5	*χ* ^2^ = 1.510	0.219
Age in years (mean ± SD) IQR	10.5 ± 3.2 6.45	11.23 ± 3.5 6.99	*t*(22) = −0.503	0.620
Age of onset (mean ± SD) QR	NA	4.5 ± 3.2 3.5	NA	NA
mRS at scan (median) Range	NA	1 0–3	NA	NA

AE, autoimmune encephalitis; mRS, modified Rankin Scale.

### Neuropsychological outcomes

**Figure 1:**
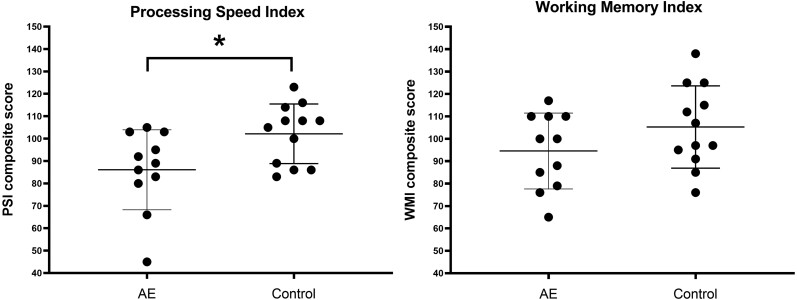
**Cognitive outcome from the neuropsychological assessments across groups**. Group comparisons were tested using independent *t*-tests. The AE group had a significantly lower average score in processing speed [*t*(21) = 2.463; *P = 0.023; Cohen’s *d* = 1.028]. Groups did not significantly differ in working memory [*t*(21) = 1.449; *P* = 0.162; Cohen’s *d* = 0.605]. AE, autoimmune encephalitis; PSI, processing speed index; WMI, working memory index; measures were obtained with the WISC-V.^44^

**Figure 2:**
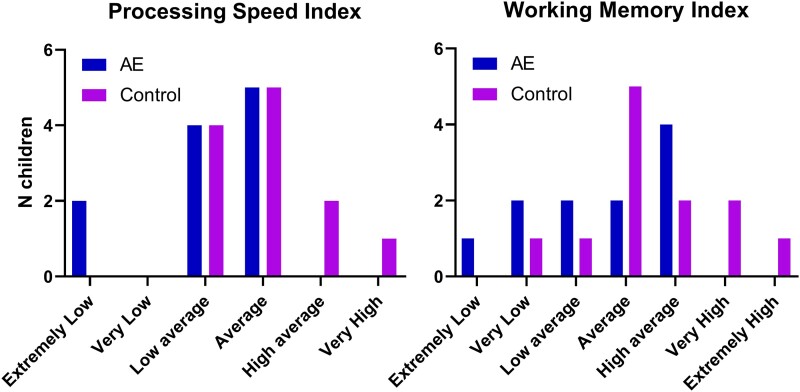
**Cognitive scores categorized according to the normative WISC-V classification.** AE, autoimmune encephalitis. Classification categories and measures were obtained with the WISC-V.^44^

One child with PAE did not complete cognitive assessments. Groups did not significantly differ in working memory (*t*(21) = 1.449; *P* = 0.162; Cohen’s *d* = 0.605), however, the PAE group had a significantly lower average processing speed (*t*(21) = 2.463; *P* = 0.023; Cohen’s *d* = 1.028; Fig. 1). Participant scores based on WISC’s age-normalized population categories are depicted in Fig. 2. No significant difference in categories was observed in processing speed (*P* = 0.414) or working memory (*P* = 0.193).

### Resting-state network analysis

#### Brain network differences between groups

**Figure 3:**
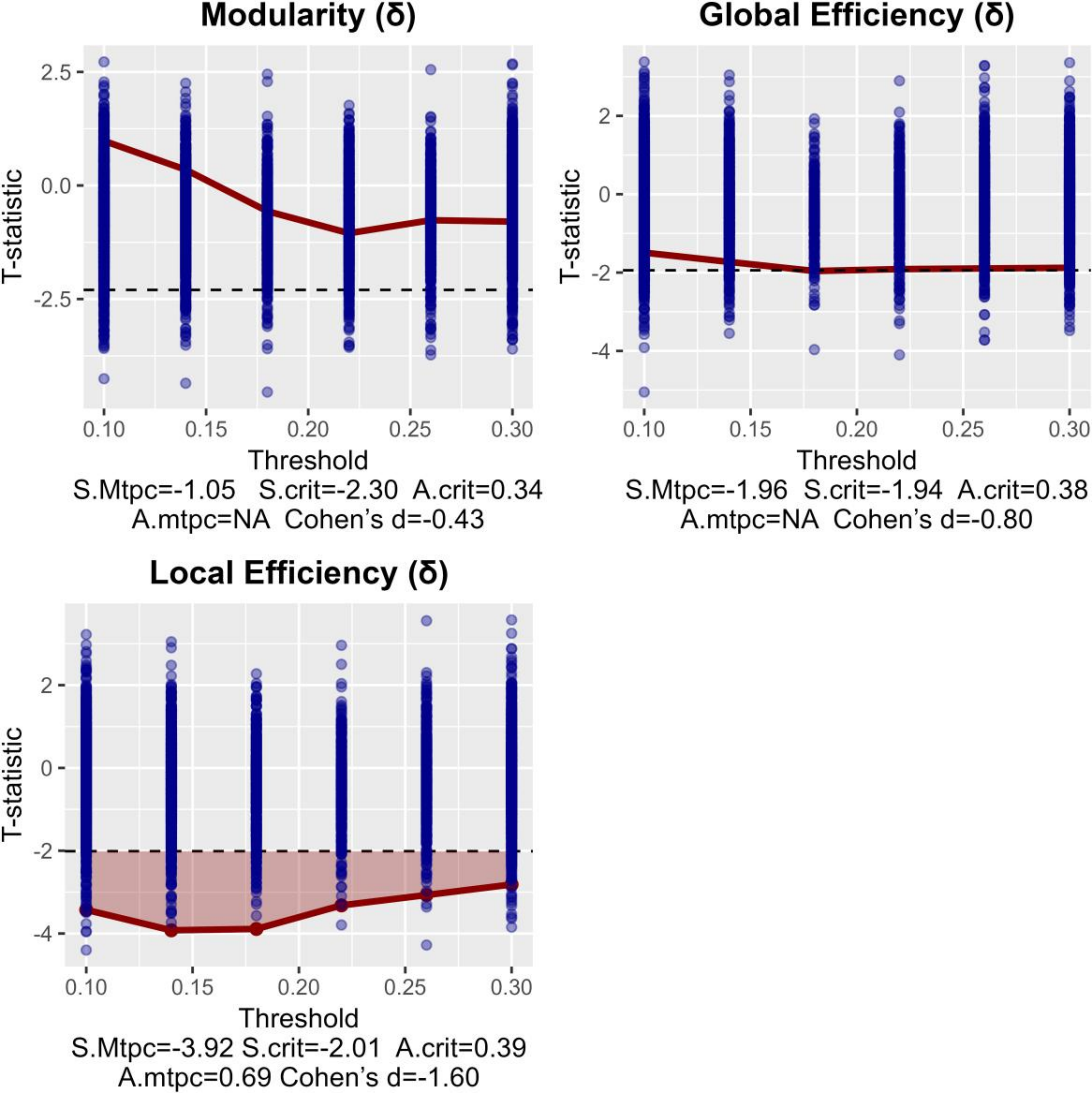
**Comparison of delta network measures (1–4 Hz) between AE and control groups, showing a significantly lower local efficiency in AE.** S.mtpc, maximum observed statistic; S.crit, critical value of the null maximum statistic; A.crit, mean of the supracritical null AUCs; A.mtpc, AUC value of supracritical cluster. Each graph depicts the network contrast of the AE group in reference to the control group. The dots are the maximum null *t* statistics of the 5000 permutations. Dots where the thick line is crossing are the observed *t* statistics. The dashed line is the critical null statistic (S.crit, top 95th percentile of the null distribution of maximum *t* statistics). The shaded areas represent clusters of observed statistics above the critical value, whose area-under-the-curve (A.mtpc) is also greater than the mean areas-under-the-curve of the supracritical permuted statistics (A.crit). This means that a lower shaded bar is a significantly lower network measure compared with the control group (at *P* < 0.05). Non-shaded bars are non-significant. The reported effect size d is only that of the threshold with the largest difference.

**Figure 4:**
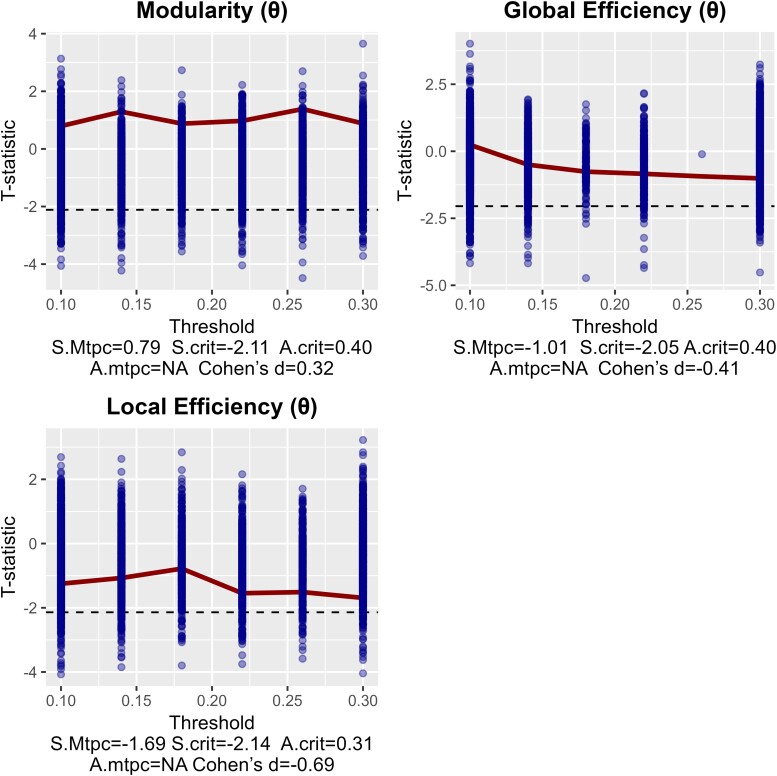
**Comparison of theta network measures (5–8 Hz) between AE and control groups, showing no significant differences** S.mtpc, maximum observed statistic; S.crit, critical value of the null max. statistic; A.crit, mean of the supracritical null AUCs; A.mtpc, AUC value of supracritical cluster. Each graph depicts the network contrast of the AE group in reference to the control group. The dots are the maximum null *t* statistics of the 5000 permutations. Dots where the thick line is crossing are the observed *t* statistics. The dashed line is the critical null statistic (S.crit, top 95th percentile of the null distribution of maximum *t* statistics). The shaded areas represent clusters of observed statistics above the critical value, whose area-under-the-curve (A.mtpc) is also greater than the mean areas-under-the-curve of the supracritical permuted statistics (A.crit). This means that a lower shaded bar is a significantly lower network measure compared with the control group (at *P* < 0.05). Non-shaded bars are non-significant. The reported effect size d is only that of the threshold with the largest difference.

Resting-state MEG recordings were obtained in all 12 controls and 12 children with PAE. Mean delta local efficiency was significantly lower in the PAE group (Fig. 3) and remained significant after correction. No significant difference was found in the theta frequency between groups (Fig. 4). The group average delta networks are depicted in Supplementary Fig. 2, highlighting regions with highest efficiency and strongest connections.

#### Network associations with cognition

The threshold at which group difference had the lowest *P*-value were theta modularity (at threshold 0.1); theta global efficiency (0.3); theta local efficiency (0.3), delta modularity (0.22), delta global efficiency (0.18) and delta local efficiency (0.14).

Models for theta modularity (*F*(2,8) = 3.348; *P* = 0.088; adjusted *R*^2^ = 0.320) and delta modularity (*F*(2,8) = 0.049; *P* = 0.952; adj. *R*^2^ = −0.234) were not significantly associated with working memory. However, theta modularity was individually associated with working memory, but this did not survive false discovery rate correction [*β* = −0.781; *t*(8) = −2.588, *P* = 0.032. All individual effect coefficients are shown in Table 2, Model 1]. Models for theta global efficiency [*F*(2,8) = 0.085; *P* = 0.919; adjusted *R*^2^ = −0.224] and theta local efficiency [*F*(2,8) = 0.029; *P* = 0.972; adj. *R*^2^ = −0.241], delta global efficiency [*F*(2,8) = 0.166; *P* = 0.850; adj. *R*^2^ = −0.200], delta local efficiency [*F*(2,8) = 0.027; *P* = 0.974; adj. *R*^2^ = −0.242] and delta modularity [*F*(2,8) = 0.181; *P* = 0.837; adj. *R*^2^ = −0.196] was not significantly associated with processing speed. The WISC-V demonstrates measurement invariance across both males and females,^44^ however, in paediatric ADEM, there is greater risk of neurological poor outcome (including intellectual difficulties) for males.^45^ Given this propensity for sex-differences in cognitive and intellectual outcomes across paediatric neuroinflammatory diseases, sex was included in these models as a predictor. To investigate the effect of the graph metrics on the WISC measures on their own, separate models were also tested excluding sex as a control variable. No significant association was observed in the exploratory analyses where the sex covariate was excluded (Supplementary Material 2; Supplementary Table 1), the theta modularity-working memory association did not reach statistical significance at *P* = 0.062.

**Table 2:** Individual effects of delta and theta graph measures on processing speed and working memory performance

		Predictor	*B*	SD error	*β*	*t*	*P*	95% confidence interval	
Model	DV							Lower	Upper
**1**	WMI	Θ *M*	−493.4	190.7	−0.78	−2.59	0.032*	−933.1	−53.7
		Sex	−12.9	9.8	−0.40	−1.33	0.221	−35.6	9.6
**2**	PSI	Θ *E*_glob_	−79.7	233.5	−0.15	−0.34	0.742	−618.2	458.8
		Sex	5.9	15.3	0.18	0.39	0.705	−29.2	41.2
**3**	PSI	Θ *E*_loc_	12.2	176.9	0.03	0.07	0.947	−395.9	420.3
		Sex	2.3	13.8	0.07	0.17	0.871	−29.4	34.05
**4**	WMI	δ *M*	−164.7	273.9	−0.23	−0.60	0.564	−791.4	476.1
		Sex	−3.1	12.2	−0.10	−0.25	0.805	−31.2	25.0
**5**	PSI	δ *M*	−62.8	293.4	−0.08	−0.21	0.836	−739.3	613.7
		Sex	1.7	13.0	0.05	0.13	0.900	−28.4	31.7
**6**	PSI	δ E.Glob	99.9	190.0	0.19	0.53	0.613	−338.3	539.2
		Sex	0.6	12.6	0.02	0.05	0.963	−28.4	29.5
**7**	PSI	δ E.Loc	−5.172	171.6	−0.014	−0.030	0.977	−400.0	390.6
		Sex	3.057	15.43	0.089	0.198	0.848	−32.53	38.64

Cognitive measures were the composite scores from the WISC-V.

DV, dependant variable; WMI, working memory index; PSI, processing speed index; *M*, modularity; *E*_glob_, global efficiency; *E*_loc_, local efficiency; Θ, theta; δ, delta.

^*^ *P* < 0.05 (fdr corrected *P* = 0.224).

Five out of the 12 models did not follow the assumption of linearity between regressors and cognition. That could not be solved using log, square root or square transformations of either dependent variables, independent variables or both. An exploratory non-parametric approach using Spearman correlations are reported in Supplementary Table 2. Delta local efficiency showed a trend towards association with WMI, but this did not reach statistical significance (*P* = 0.067).

## Discussion

This study used advanced network analyses of MEG recordings to investigate whether disrupted functional connectivity was associated with cognitive outcomes in PAE. Resting-state connectivity in delta and theta frequency bands were analysed in children with PAE and similarly aged typically developing controls. Metrics describing the organization of functional networks were included in regression models in association with processing speed and working memory, two domains known to be affected in PAE. Local efficiency within delta networks was lower on average in PAE compared with controls (and trended towards a correlation with WMI). Modularity in theta networks was associated with lower working memory in PAE. This is the first study to demonstrate feasibility and validity of MEG-based functional connectivity analyses in a cohort of children with PAE.

In this current study, a proportion of PAE patients had an identifiable cognitive impairment in processing speed (18.2%) and working memory (27.2%), consistent with other studies.^3,6,44,45^ It is important to note that the PAE cohort was considered to have a ‘good’ outcome on average (mRS ≤ 24), with only one child having moderate disability (mRS = 3). Processing is among the most common difficulties in paediatric NMDARE and ADEM^3,46-48^ with patients likely to struggle with responding rapidly to task-relevant information, even years after disease onset. The use of resting-state neuroimaging in this population is therefore highly relevant, being easier to undertake by children who may struggle with the demands of an interactive task or paradigm.

In delta frequency, neither modularity nor global efficiency differed from controls, but local efficiency was significantly lower in PAE. This suggests that while an efficient topology and modularity of the overall functional brain network is preserved, the local topology is altered. When considering brain regions that form local neighbourhoods (not necessarily local in the sense of spatial location in the brain), information transmission between these regions is less efficient than would typically be expected in the healthy brain. The delta band specificity may be linked to commonly reported abnormalities in AE patient EEG, such as extreme delta brush and generalized rhythmic delta activity.^24,25^ EEG slowing of brain activity (delta frequency range) is frequently noted in ADEM^49^ and NMDARE.^23^ This present study suggests delta activity may be affected long-term in PAE and could represent a good candidate biomarker for future research.

Increased theta modularity was associated with lower working memory. Such a result, if replicated in larger studies, suggests that theta activity tends to form individual and distinct modules within the network, making it harder for children with PAE to keep information in mind and manipulate it. This may reflect a narrowing of a normally widely distributed network underlying working memory functioning. Recent research has shown that theta connectivity in specific edges were correlated negatively to working memory in paediatric temporal lobe epilepsy^50^ supporting our observation that alterations in typical patterns of theta activity may interfere with working memory function in children with neurological disease. Theta modularity may represent a sensitive measure to detect differences in working memory in PAE.

### Limitations

Sample size (a result of the COVID pandemic, condition rarity and single-centre recruitment), is the main study limitation. Furthermore, the time between disease onset and study time was on average 7 years and up to 15 years; this long interval may have influenced the results—longitudinal studies with more regular and shorter interval assessments would more accurately track outcomes.

The present findings must therefore be interpreted with caution and cannot be extrapolated to the entire population of children with PAE. However, they do offer compelling preliminary findings and provide direction for future larger-scale studies. Additionally, this ‘real-world’ cohort was heterogeneous in terms of subtypes of AE and was too small to be subdivided. This limits the interpretation of the results, as the majority of the patients in the study were diagnosed with ADEM; it remains to be verified whether the present findings apply more to ADEM than to other AE subcategories (NMDARE, etc.).

There were also manual processes, such as manual edits to correct for MRI artefacts, or manual fiducial positioning of the MEG coordinates along the MRI scans, which may have introduced a level of bias and to mitigate this, the guidance detailed in the FreeSurfer online resources was followed: (https://surfer.nmr.mgh.harvard.edu/fswiki/FsTutorial/TroubleshootingData).

The change of MRI scanner over the course of recruiting the cohort raises the possibility that scanner differences may have biased MEG source estimation. However, given there is remarkable consistency between both native MRI and template MRI source localization of MEG connectivity^51^ the impact is likely to be minimal. The two MRI scanners used in the current study were from the same vendor (Siemens), and research radiography staff worked with the study team to ensure MRI protocols was identically matched across the two scanners.

Although maxfiltering was not used on MEG resting-state recordings, and no principal component analysis was used, an ICA was deemed necessary to obtain a sufficiently clean data for further analysis. This involves the risk of the phase producing spurious phase changes as documented in some EEG simulations.^52^ The extent to which such distortion impacts MEG data and amplitude envelope correlation is however unknown, and signal decomposition in paediatric MEG data still appear to be the most efficient way to address artefacts in the paediatric cohorts.

## Conclusion

This study reinforces previous findings that children with PAE have ongoing residual cognitive difficulties in the long-term, with lower performance in processing speed compared with typically developing children, despite being classified as having a ‘good’ medical outcome. Resting-state MEG recordings indicated lower local efficiency within delta frequency networks of children with PAE and those higher levels of modularity within the theta-frequency resting-state network may be associated with lower working memory. Future studies will benefit from larger sample size and newer neuroimaging approaches, for example using OPM-MEG, guided by the measures investigated in the present study. The preliminary data presented supports MEG as an appropriate and feasible technique to characterize functional dysconnectivity in PAE.

## Supplementary Material

fcae248_Supplementary_Data

## Data Availability

The data that support the findings of this study are available from the corresponding author, upon reasonable request. All codes generated for analysis are detailed in Supplementary Material 3.
