# Supplementary material for: Examining cognition and brain networks using magnetoencephalography in paediatric autoimmune encephalitis and acute disseminated encephalomyelitis: a preliminary study
Source: Brain Commun. 2024 Aug 8;6(4):fcae248. doi: 10.1093/braincomms/fcae248 (PMC11316206; doi:10.1093/braincomms/fcae248)
Supplement: fcae248_Supplementary_Data [file fcae248_supplementary_data.docx]

**Supplementary material**

**Supplementary Figure 1** **Delta connectivity matrix estimated in a participant** AEC = Amplitude Envelope Correlation. The figure was produced with Brainstorm.^1^ The matrix depicts the level of connectivity between each of the 68 brain regions parcellated with the Desikan-Killiany atlas.


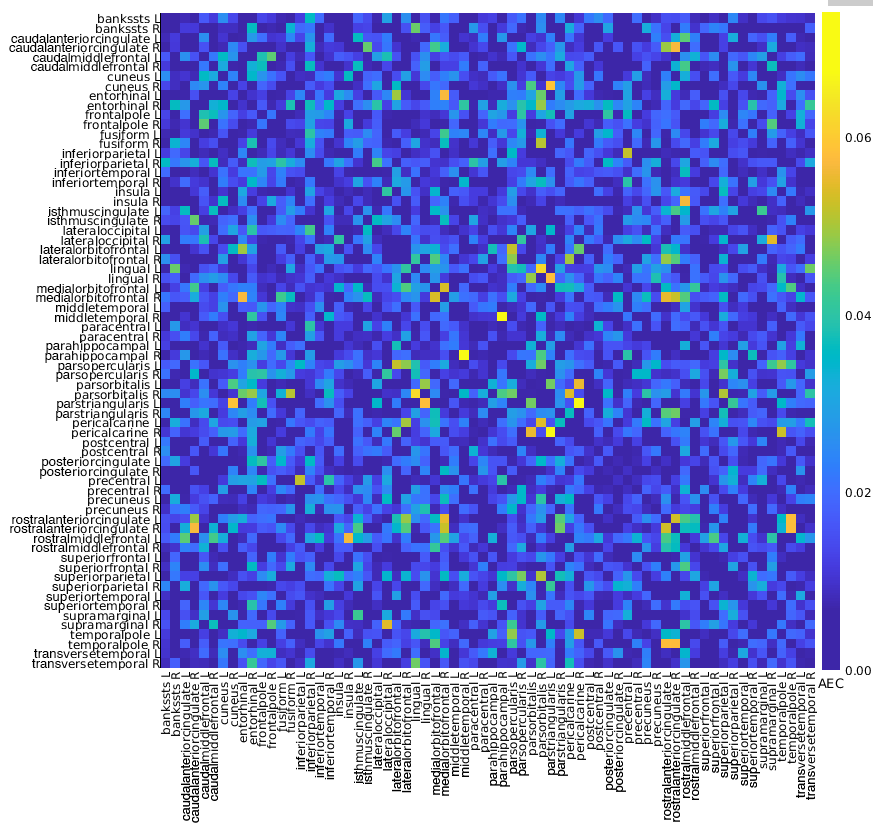


**Supplementary Figure 2** **Depiction of average delta networks in Autoimmune Encephalitis and Control groups, highlighting regions where efficiency is the highest and connections are the strongest** AE= Autoimmune encephalitis; C = Controls. 3D surfaces are based on the fsaverage FreeSurfer template. Node size and brightness represent the group average local efficiency per region, and edge thickness and brightness represent the group average strength of the connectivity (not relatively to the other group, on the same scale). The network was thresholded at 14%. The figure was produced using the NeuroMArVL web app (<https://immersive.erc.monash.edu/neuromarvl/>).


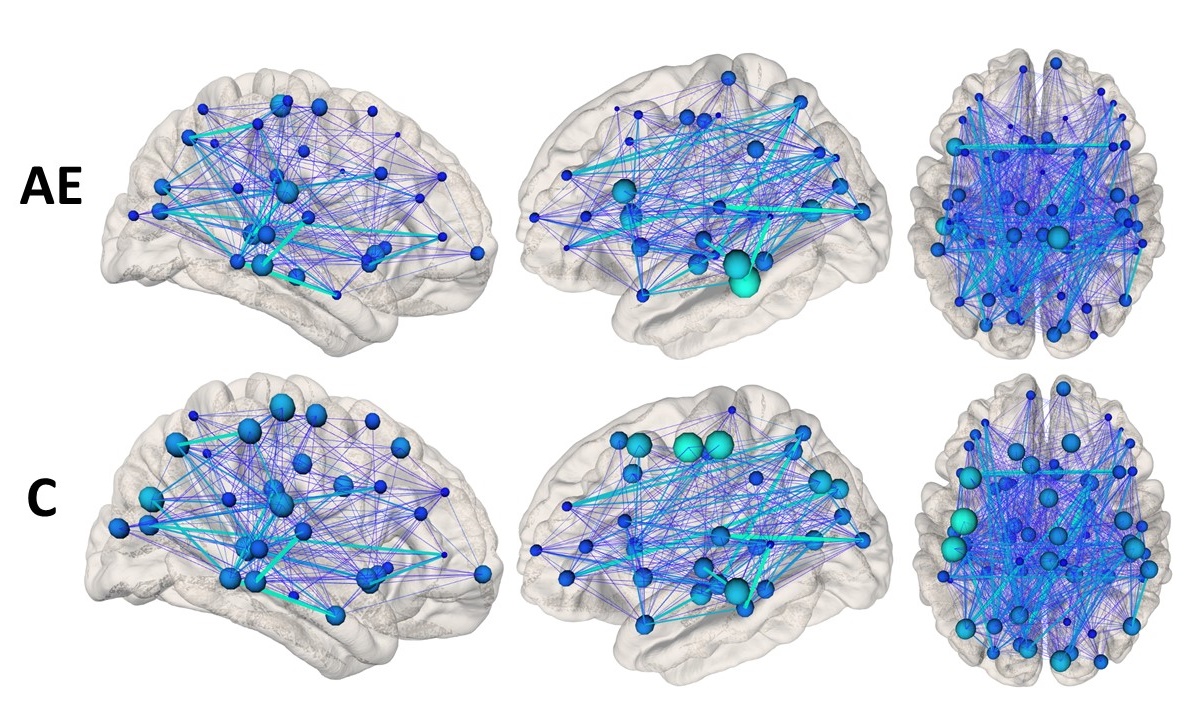


**Supplementary material S1**. **Post-hoc analysis checking for difference in raw functional connectivity when graph networks significantly differed**

For significant differences, a post-hoc exploratory analysis was run to verify a potential difference in overall raw functional connectivity (overall FC), compared across groups using nonparametric permutation tests (t-test with threshold 3.1, two-tailed, 5000 permutations, significant at *p=*.05, component size=extent) in the *Network Based Statistic* toolbox.^2^ The point of such post-hoc analysis is to verify whether the network organization differences may simply be explained by the overall difference in FC or, on the contrary, remain different regardless of this overall connectivity contrast. That is because low overall FC can introduce spurious connections within proportional thresholds and in turn influence network metrics.^3,4^ If overall FC differed between groups, a strategy proposed by van den Heuvel *et al.*^4^ was followed if applicable to the data, by establishing overall FC-matched subgroups and rerunning the graph metrics analyses.

No difference in overall functional connectivity was observed across groups in either frequency band.

**Supplementary material S2. Network associations with cognition without the sex covariate**

Results of this analysis are shown in Supplementary table 1 (below). The regression model for theta modularity was not significantly associated with Working memory performance (*F*(1,9)=4.552; *p=*.062; adjusted *R²=*0.262). The regression models for theta global efficiency (*F*(1,9)=0.017; *p=*.898; adjusted *R²=*-0.109) and theta local efficiency (*F*(1,9)=0.033; *p=*.861; adjusted *R²=*-0.107) did not significantly predict processing speed performance. The regression models for delta modularity did not significantly predict working memory performance (*F*(1,9)=0.332; *p=*. 579; adjusted *R²=*-0.071) or processing speed performance (*F*(1,9)=0.092; *p=*.769; adjusted *R²=*-0.100). The regression model for delta global efficiency (*F*(1,9)=0.370; *p=*.558; adjusted *R²=*-0.067) and delta local efficiency did not significantly predict processing speed performance (*F*(1,9)=0.016; *p=*.901; adjusted *R²=*-0.109).

**Supplementary Table 1**

*Individual effects of delta and theta graph measures on Processing Speed and Working Memory Performance without sex as a control variable*

|  |  | **Predictor** | ***B*** | **sd error** | ***β*** | ***t*** | ***p*** | **Confidence interval 95%** | |
| --- | --- | --- | --- | --- | --- | --- | --- | --- | --- |
| **Model** | **DV** |  |  |  |  |  |  | **Lower** | **Upper** |
| **1** | WMI | Θ *M* | -366.36 | 171.71 | -0.579 | -2.134 | .062 | -754.80 | 22.08 |
| **2** | PSI | Θ *E_glob_* | -22.82 | 174.43 | -0.043 | -0.131 | .898 | -417.40 | 371.75 |
| **3** | PSI | Θ *E_loc_* | 26.55 | 146.40 | 0.060 | 0.181 | .861 | -304.62 | 357.72 |
| **4** | WMI | δ *M* | -137.85 | 239.22 | -0.189 | -0.576 | .579 | -678.99 | 403.29 |
| **5** | PSI | δ *M* | -77.48 | 255.42 | -0.101 | -0.303 | .769 | -655.28 | 500.33 |
| **6** | PSI | δ E.Glob | 102.91 | 169.20 | 0.199 | 0.608 | .558 | -279.86 | 485.67 |
| **7** | PSI | δ E.Loc | 16.06 | 126.68 | 0.042 | 0.127 | .902 | -270.52 | 302.63 |

PSI processing speed index WMI working memory index

**Supplementary Table 2**

*Exploratory analysis of relationships between predictor variables and dependent variables that could not be tested in the regression, using Spearman correlation (linear relationship not assumed).*

| Relationship | S | *p*-value | *rho* |
| --- | --- | --- | --- |
| Theta modularity + PSI | 263.1 | 0.563 | -0.196 |
| Theta global efficiency + WMI | 234.1 | 0.851 | -0.064 |
| Theta local efficiency + WMI | 195.7 | 0.747 | 0.110 |
| Delta global efficiency + WMI | 218 | 0.979 | 0.009 |
| Delta local efficiency + WMI | 94.6 | 0.067 | 0.570 |

PSI processing speed index WMI working memory index

**Supplementary information S3. Codes generated for the analysis**

**S5.1 Fieldtrip preprocessing pipeline in MATLAB for resting-state recordings:**

%Define the name of the subject and the fif file
subj.name = 'sub-XXXX';
subj_path = [path_to_fif_recordings]
recording_file = sprintf('[path_to_fif_recording]/%s_task-resting_meg.fif',subj.name, subj.name);

%create dataset from fif file
cfg = [];
cfg.dataset = recording_file;
cfg.channel = {'MEGGRAD'};
cfg.continuous = 'yes';
cfg.lpfilter = 'yes';
cfg.lpfiltord = 4;
cfg.lpfreq = 70;
data = ft_preprocessing(cfg);

%create epochs of 10 seconds, with DC component removal
cfg = [];
cfg.length = 10;
dataepoch = ft_redefinetrial(cfg,data);

cfg = [];
cfg.demean = 'yes';
dataepoch = ft_preprocessing(cfg,dataepoch)

%Independant component analysis (ICA)
cfg = [];
cfg.channel = {'MEGGRAD'};
cfg.method = 'runica';
comp = ft_componentanalysis(cfg, dataepoch);

%View the components
cfg = [];
cfg.layout = 'neuromag306planar.lay';
cfg.compscale = 'local';
cfg.continuous= 'no';
ft_databrowser(cfg, comp);

% remove undesired components
cfg = [];
cfg.component = [array of undesired component numbers] ;
rejectedcomp = cfg.component;
dataclean = ft_rejectcomponent(cfg, comp, dataepoch);
numel(cfg.component)

%remove bad trials then bad channels summary with the fieldtrip GUI:
cfg = [];
cfg.method = 'summary';
cfg.channel = {'MEGGRAD'};
dataclean = ft_rejectvisual(cfg, dataclean);

cfg = [];
cfg.method = ‘channel’;
cfg.channel = {'MEGGRAD'};
dataclean = ft_rejectvisual(cfg, dataclean);

save(dataclean)

**S5.2 Matrices thresholding**

%This scripts loads all matrices extracted from Brainstorm, applies thresholds to all of them, and makes folders with all matrices for each threshold in their respective delta or theta files. It uses the Brain Connectivity Toolbox (v.2019-03-03).
%it also converts them into .csv to run in R

%search for all matrices available (example: ‘sub-0000.theta.mat’. Each matrix object handle was named after the frequency)
search = dir(fullfile('[path_to_matrices]/sub-*.* _matrix.mat'));

for j = 0.10:0.04:0.30 %for each threshold

for k = 1:length(search) %for each subject

filename = search(k).name;
 load(fullfile(search(k).folder, filename))

subjname = strfind(filename, '.m');
 subjname = filename(1:subjname-1); %extract file name

%attributes right frequency by reading filename and removes mat extension
 split_filename = split(filename,["-",".","_"])
 number = str2num(split_filename{2})
 frequency = split_filename{3}

if frequency == 'delta'

delta(delta<=0) = 0; %remove negative correlations
 delta_n = weight_conversion(delta, 'normalize'); %normalize matrix
 delta_t = threshold_proportional(delta_n, j); %apply threshold

elseif frequency == 'theta'

theta(theta<=0) = 0; %remove negative correlations
 theta_n = weight_conversion(theta, 'normalize'); %normalize matrix
 theta_t = threshold_proportional(theta_n, j); %apply threshold

end

%creates (if absent) each threshold folder, and stores matrices accordingly

if ~exist(sprintf('[my_path]/%s_matrices/', frequency), 'dir')

mkdir(((sprintf('[my_path]/%s_matrices/', frequency)))); end

if ~exist(sprintf('[my_path]/%s_matrices/threshold_%2.f/', frequency, j), 'dir')

mkdir(((sprintf('/[my_path]/%s_matrices/threshold_%.2f/', frequency, j)))); end

%saves matrix in appropriate folder

if frequency == 'delta';

csvwrite(sprintf('[my_path]/%s_matrices/threshold_%.2f/%s_t%.2f.csv', frequency, j, subjname, j), delta_t) % to make sure it's rounded to 2 decimals: %.2f

elseif frequency == 'theta';

csvwrite(sprintf('[my_path]/%s_matrices/threshold_%.2f/%s_t%.2f.csv', frequency, j, subjname, j), theta_t)

end

end

end

**S5.3 BrainGraph g.list preparation in R:**

#This script reads into all the matrices extracted from Brainstorm, at each desired threshold, and structures them according to the brainGraph structure (g.list) that can be used for the MTPC algorithm

library(tidyverse)
library(abind)
library(brainGraph)
library(igraph)

frequency = "delta"

#reads all threshold folders, and in each folder, reads all matrix files
directorynames = list.dirs("matrices", recursive = TRUE)
directorynames = grep(frequency, directorynames, value = TRUE)
directorynames = directorynames[2:7]

filenames = list()

for(i in 1:length(directorynames))
{
filenames[i] <- list(list.files(directorynames[i], pattern="*.csv", full.names=TRUE))
}

#imports DK atlas but reorder labels according to the brainstorm label order, that is, alphabetically rather than per lobe:

atlasreorder = function(dkatlas)
{

#for each name, it will move the hemisphere letter of the labels to the end of the string, so they can be rearranged alphabetically as in the brainstorm's Desikan Killiany order

for(i in 1:length(dkatlas$name.full))

{
 if (startsWith(dkatlas$name.full[i], "L") == TRUE)

{
 dkatlas$name.full[i] = sub("L ", "", dkatlas$name.full[i])
 dkatlas$name.full[i] = paste0(dkatlas$name.full[i], " L")
 } else {
 dkatlas$name.full[i] = sub("R ", "", dkatlas$name.full[i])
 dkatlas$name.full[i] = paste0(dkatlas$name.full[i], " R")
 }

}
 dkatlas = arrange(dkatlas, name.full, hemi)
}

dkatlas = dk
dkatlas = as_atlas(atlasreorder(dkatlas))

#Prepares g.list
#the g.list will contain each list of matrices per threshold
#(6 thresholds, meaning 6 graphlists listed in the g.list)

g.list = list()

for (t in 1:length(filenames)) #for each threshold t
{

#Prepares 3D array (containing all subj matrices at threshold t)

mlist = array(dim=c(68,68,0))

#Prepares subj-names list and group-names list that will be assigned to corresponding matrices

gnames = c()
 grpNames = c()

#imports each subject matrix, and stores each in "filenames" list

for(i in 1:length(filenames[[t]])) #for each subject i

{
 #extracts subj name from file path, adds it to subj-name list
 #Note: this works only with the following ID format: “sub-[4digits]”

name = substr(filenames[[t]][i], 40, 47)
 gnames = c(gnames, name)

#assigns group in list according to subj ID

if(as.numeric(gsub("sub-","",name)) < 5000) {
 grpNames = c(grpNames, "Case") }

else {
 grpNames = c(grpNames, "Control") }

#imports matrix csv of subject, assigns labels
 #according to reordered atlas:

matrix = read_delim(filenames[[t]][i], delim = ",",

#gives every column its corresponding atlas name
 col_names=as.character(dkatlas$name))

#adds column with labels and turn it in row names
 matrix = rowid_to_column(matrix, "labels")
 matrix$labels = dkatlas$name
 matrix = column_to_rownames(matrix, "labels")

#converts imported matrix to numeric R matrix:
 matrix = data.matrix(matrix, rownames.force = NA)

#adds matrix to the 3D array
 mlist <- abind(mlist, matrix, along = 3)

}

#Once 3D matrix for each threshold is done, make a "graphlist object" that will assign to each matrix its name, group, labels, etc, then adds it to a g.list grand list

graphlist = make_brainGraphList(mlist,

atlas = "dk", type = "observed", level = "subject",

#Note: use dk, you don't need to use dkatlas, since you've given the labels in the matrix col_names it will automatically match them with the dk labels in the order you've given

set.attrs = TRUE, modality = "meg", weighting = "auc", mode = "undirected", weighted = TRUE,

#extract threshold from filename
 threshold = as.numeric(substr(filenames[[t]][1], 35, 38)),
 gnames = gnames, grpNames = grpNames

)

#then adds graphlist to g.list
 g.list[[t]] = graphlist

}

#save g.list for that frequency
save(g.list,file=paste0("g.list_", frequency, ".rds"))

**S5.4 Multi-threshold permutation correction script**

#This script runs the multiple threshold permutation correction algorithm, based on a g.list containing the list of graphs for each treshold. It runs one mtpc analysis per desired graph metrics, and stores all of them into a file.

library(tidyverse)
library(brainGraph)
library(igraph)
library(data.table)

frequency = "delta"
load(file=paste0("g.list_", frequency, ".rds"))

#makes thresholds list for mtpc

thresholds = c()

for(t in 1:length(g.list)) {
 thresholds = c(thresholds, g.list[[t]]$threshold)
 }

#Prepares variables to be used in covariate table
#(Study.ID, Group etc.)
# Note [24.05.2024]: It seems that in more recent versions of brainGraph, reading content of the graphs fail ($df no longer readable). However, installing brainGraph via devtools::install_github('cwatson/brainGraph') seems to fix the issue.

Study.ID = c()

Group = c()

for(s in 1:length(g.list[[1]]$graphs)) {

Study.ID = c(Study.ID, summary(g.list[[1]]$graphs[[s]])$df[[13,2]])

if (summary(g.list[[1]]$graphs[[s]])$df[[14,2]] == "Case")

{ Group = c(Group,1) }
 else
 { Group = c(Group,0) }

}

#create a covariate table depending on what you want ton include:
#mtpc always accounts for every covariate

covars = data.table("Study.ID" = Study.ID, "Group_Patient" = Group)

#Set design contrast matrix for a t-test (basic difference)
#each column refers to a column in covars (based on bG manual, dummy coded, 8.3.4)

contrasts = matrix(c(0,1), nrow = 1, ncol = 2, byrow = TRUE,
 dimnames = list(c("Control > Patient")))

#Select graph measures (A.1.3 of bG manual). At level graph:
# "mod.wt" -- modularity,
# "E.global.wt" -- global efficiency,
# 'E.local.wt" -- mean local efficiency:

measure = c("mod.wt", "E.global.wt", "E.local.wt")

#fix for mtpc by Chris Watson for R > 4.0.0
#https://groups.google.com/g/braingraph-help/c/_Zix0nwVamc/m/Ap_lO9ThBgAJ

qr.matrix <- function(x, ...) { qr.default(x, ...) }

#Run mtpc analysis for each measure and store outputs into a list:

mtpc_list = list()
for(m in 1:length(measure)) {

#for each measure, run MTPC with defined parameters
 mtpc = mtpc(g.list, thresholds, covars, measure[m], contrasts,
 con.type = "t", level="graph", N = 5000, alternative = "less", coding ="dummy")

#assign name to it
 mtpc = list(mtpc); names(mtpc) = measure[m]

#stores it in list
 mtpc_list = append(mtpc_list, mtpc)

}

#to look into output individually: summary(mtpc_list$mod.wt)
#to plot the output: plot(mtpc_list$mod.wt)

#Cohen's d can be computed by doing
best_t = which.min(mtpc_list$mod.wt$DT[,p])
max(mtpc_list$mod.wt$DT[best_t,gamma])/sigma(mtpc_list$mod.wt$res.glm[[best_t]])

#(gets which threshold has lowest p-value difference, and gets difference between means: i.e. gamma contrast; then divides that contrast by its residual sd, i.e. root mean squared error RMSE; cf. bGmanual)

#save mtpc_list
save(mtpc_list, file=paste0("mtpc_list_", frequency, ".rds"))

**References**

1. Tadel F, Baillet S, Mosher JC, Pantazis D and Leahy RM. Brainstorm: a user-friendly application for MEG/EEG analysis. *Computational intelligence and neuroscience* 2011; 2011: 1-13.
2. Zalesky A, Fornito A and Bullmore ET. Network-based statistic: identifying differences in brain networks. *Neuroimage* 2010; 53: 1197-1207.
3. Hallquist MN and Hillary FG. Graph theory approaches to functional network organization in brain disorders: A critique for a brave new small-world. *Network neuroscience* 2018; 3: 1-26.
4. van den Heuvel MP, de Lange SC, Zalesky A, Seguin C, Yeo BT and Schmidt R. Proportional thresholding in resting-state fMRI functional connectivity networks and consequences for patient-control connectome studies: Issues and recommendations. *Neuroimage* 2017; 152: 437-449.
